# Variation of perioperative plasma mitochondrial DNA correlate with peak inflammatory cytokines caused by cardiac surgery with cardiopulmonary bypass

**DOI:** 10.1186/s13019-015-0298-6

**Published:** 2015-06-24

**Authors:** Chaoyi Qin, Ruiqi Liu, Jun Gu, Yajiao Li, Hong Qian, Yingkang Shi, Wei Meng

**Affiliations:** 1 Department of Cardiovascular Surgery, West China Hospital, Sichuan University, Chengdu, 610041 People's Republic of China; 2 Department of Burns and Plastic Surgery, West China Hospital, Sichuan University, Chengdu, 610041 People's Republic of China; 3 Department of Cardiology, West China Hospital, Sichuan University, Chengdu, 610041 People's Republic of China

**Keywords:** Cardiopulmonary bypass, Mitochondrial DNA, Inflammatory cytokine

## Abstract

**Backgroud:**

Cardiac surgery with cardiopulmonary bypass (CPB) may cause inflammatory responses, which can deteriorate the outcomes. Inflammatory cytokines, such as tumor necrosis factor (TNF)-α, interleukin (IL)-6,–8 and -10, can act as both the effector and the predictor for post-operative inflammatory responses. Plasma mitochondrial DNA (mtDNA) was found as a pro-inflammatory agent recently, which was released when cells were insulted.

**Methods:**

In the present study, we included 38 patients undergoing coronary artery bypass graft (CABG) to analyze their perioperative plasma mtDNA and levels of inflammatory cytokines. Blood samples were collected before aortic cross-clamping (T1), at the end of CPB (T2), 6 h post-CPB (T3), 12 h post-CPB (T4), and 24 h post-CPB (T5). Rt-PCR and specific ELISA kits were used to quantify the plasma mtDNA and inflammatory cytokines, respectively. Bivariate correlations analysis was used to check the correlations between plasma mtDNA and inflammatory cytokines respectively.

**Results:**

Results shown that plasma mtDNA elevated significantly at T2 and peaked at T4. Furthermore, plasma TNF-α, IL-6 and IL-8 levels significantly increased at T2 and peaked at T3 while IL-10 elevated and peaked at T2. Bivariate correlations analysis showed that the peak plasma mtDNA were positively correlated with the peak TNF-α (*r* = 0.677, *P* < 0.001), the peak IL-6 (*r* = 0.706, *P* < 0.001), the peak IL-8 (*r* = 0.584, *P* < 0.001) and the peak IL-10 (*r* = 0.565, *P* < 0.001).

**Conclusion:**

We found that plasma mtDNA might play a key role in CPB-induced post-operative inflammatory responses.

## Background

The first case of extracorporeal circulation (ECC) was reported by Gibbon JH in 1954 to successfully save a patient with atrial septal defect (ASD) [1]. In 1955, Kirklin *et al.* combined the ECC with the oxygenator in intra-cardiac surgery and since then, the technique of cardiopulmonary bypass (CPB) was developed to be the key process in modern cardiothoracic surgery [2]. While the CPB helping to bypass circulation out of the heart and lung during the operation, the abnormal status of circulation and the ischemia/reperfusion injury (I/R injury) may cause inflammatory responses [3]. It is well known that most of postoperative complications are related to the systemic inflammatory response syndrome (SIRS) and many studies demonstrated that inflammatory mediators, like tumor necrosis factor (TNF)-α, interleukin (IL)-6, IL-8 and IL-10, increased significantly after cardiac surgery with CPB [4–7]. Although many anti-inflammatory agents and coated circuits were investigated to reduce the inflammatory responses after CPB, we still need a more efficient way to eliminate the inflammatory responses and then protect the secondary damage to the heart and other organs [8, 9].

Human mitochondrial DNA (mtDNA) consist of 16569 nucleotide bases and take responsible for encoding 13 polypeptides of the electron transport chain, 22 transfer RNAs, and 2 ribosomal RNAs [10]. mtDNA was released into circulation and trigger inflammatory responses when cells were dealing with harmful insults [11]. With the pro-inflammatory CpG motif, mtDNA acts as a damage-associated molecular patterns (DAMPs) [12]. It is documented that mtDNA played a pro-inflammatory role in several diseases. Plasma mtDNA was elevated remarkably in trauma patients, comparing to volunteers [13]. Recently, a study based on multiple cohorts showed that mtDNA can improve risk prediction and there is a tight relationship between elevated plasma mtDNA level and 28-day mortality [14].

Given the pro-inflammatory features of mtDNA and series of inflammatory responses after CPB, we hypothesized that mtDNA may act as a pro-inflammatory factor after cardiac surgery with CPB and play a key role in post-CPB inflammatory responses with other inflammatory factors, such as TNF-α, IL-6, IL-8 and IL-10.

## Methods

### Patients

Thirty-eight patients were included from January 2014 to August 2014, who were admitted to the Department of Cardiovascular Surgery, West China Hospital, requiring coronary artery bypass graft (CABG). Medical histories of endocarditis, diabetes, hypertension, neurological diseases, psychiatric diseases, infectious diseases, and post-surgical acute renal failure and low cardiac output syndrome were designed as the excluding criteria. All patients had normal function of kidney, liver and lung prior to surgery. Informed consents were signed by every patients. CABG with CPB and postoperative standard care in cardiac intensive care unit (CICU) were performed successfully for all patients. The study was conducted following the Declaration of Helsinki and registered in the research committee at the Sichuan University.

### Blood samples collection

Blood samples were collected in EDTA-coated blood collection tube before aortic cross-clamping (T1), at the end of CPB (T2), 6 h post-CPB (T3), 12 h post-CPB (T4), 24 h post-CPB (T5). The whole blood was centrifuged at 1000 rpm/min for 15 min at 4° and subsequently supernatant was collected as plasma. Samples of plasma were stored in -80° freezer and ready for rt-PCR and ELISA.

### DNA isolation and rt-PCR for mtDNA

The whole plasma DNA was isolated from plasma using the DNeasy Blood and Tissue Kit (#69504, Qiagen). Briefly, 50 μL plasma samples were added to50 μL phosphate buffered saline (PBS) and then centrifuged at 16000 g for 15 min at 4°. 90 μL of supernatant were kept for the next procedures. The rest procedures were performed according to the manufacture’s protocol. At the last step, 200 μL elution buffer were added to resolve the plasma DNA.

Plasma mtDNA levels were measured by SYBR-green dye-based rt-PCR assay using a PRISM 7300 sequence detection system. The primer sequences were human NADH dehydrogenase 1 gene (mtDNA): forward CGAGCAGTAGCCCAAACAAT, reverse TGTGATAAGGGTGGAGAGGTT. Plasmid DNA with complementary DNA sequence for human mtDNA was obtained from ORIGENE (SC101172, USA). Concentration of plasma mtDNA were converted to copy number via a DNA copy number calculator (http://cels.uri.edu/gsc/cndna.html; University of Rhode Island Genomics and Sequencing Center). Plasmid DNA were diluted in 10-fold serial dilutions and measured as standard curve [14].

All samples were measured with standards at the same time. Plasma mtDNA levels were shown in copies per microliter of plasma according to the following formula:

$$ \mathrm{c} = \mathrm{Q}\ *\ {\mathrm{V}}_{\mathrm{DNA}}/\ {\mathrm{V}}_{\mathrm{PCR}}*\ 1\ /\ {\mathrm{V}}_{\mathrm{ext}} $$

where c is the concentration of plasma mtDNA (copies/μL); Q means the quantity of DNA measured by rt-PCR; V_DNA_ means the total volume of plasma DNA solution obtained from the extraction, 200 μL in this study; V_PCR_ means the volume of plasma DNA solution for rt-PCR, 1 μL in this study; V_ext_ means the volume of plasma used for the extraction, 50 μL in this study.

### Measurement of cytokines

Plasma TNF-α, IL-6, IL-8 and IL-10 were measured by enzyme-linked immunosorbent assay (ELISA) kit (Solarbio, China). All procedures were followed standard protocols (included in the ELISA kits). Spectrophotometry was used to detect the intensity of the transmitted light. Data was expressed in picogram per mL.

### Statistically analysis

All descriptive data were shown as mean ± standard error of the mean (SEM). Multiple comparisons were analyzed by one-way ANOVA followed by Bonferroni’s test. The Pearson correlation coefficient test were performed. *P* < 0.05 was considered to statistically significant.

## Results

### Baseline information

Patient basic characteristics, surgery information and laboratory data were presented in Table 1. 38 patients were included and performed CABG successfully. During the surgery, the average of aortic cross-clamping time is (68 ± 21) min and the average CPB time is (96 ± 27) min. On admission, we collected the baseline levels of serum creatinine, hemoglobin, total cholesterol (TC), high density lipoprotein (HDL), low density lipoprotein (LDL), triglycerides (TG), glucose and hematocrit (Hct).

**Table 1:** Baseline information

Characteristics	*N* = 38
Age (years)	49.4 ± 12.7
Women	24 (63.2 %)
BMI (kg/m^2^)	23.2 ± 3.2
Emergency	7 (18.4 %)
Urgent	9 (23.7 %)
Elective	22 (57.9 %)
STEMI	5 (13.2 %)
NSTEMI	8 (21.1 %)
Stable angina	14 (36.8 %)
Unstable angina	11 (28.9 %)
Surgery information	
Aortic Cross-clamping Time (min)	68 ± 21
CPB Time (min)	96 ± 27
Laboratory data	
Serum Creatinine (μmol/L)	0.6 ± 0.2
Hemoglobin (g/L)	130.3 ± 11.7
Total Cholesterol (mg/dL)	198.2 ± 35.9
HDL (mmol/L)	1.1 ± 0.2
LDL (mmol/L)	2.9 ± 0.6
TG (mmol/L)	1.5 ± 0.4
Glucose (mg/dL)	140.1 ± 34.8
Hct (%)	39.4 ± 3.9

Variables are presented as mean ± SEM or as numbers (%). *CPB* cardiopulmonary bypass, *BMI* Body mass index, *STEMI* ST elevated myocardial infarction, *NSTEMI* Non- ST elevated myocardial infarction, *HDL* high density lipoprotein, *LDL* low density lipoprotein, *TG* Triglycerides, *Hct* Hematocrit

### Changes of perioperative plasma mtDNA levels

**Fig. 1:**
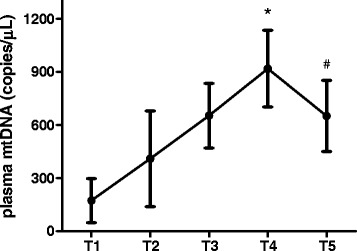
Perioperative plasma mtDNA in CABG with CPB. Plasma mtDNA levels after the end of CPB were significantly higher than T1. The peak value of plasma mtDNA appeared at T4. Level of plasma mtDNA at T5 was lower than T4, but still higher than T1. *P < 0.05 vs. T1. #*P* < 0.05 vs. T4

Plasma mtDNA levels at different time points were measured by rt-PCR. Given that much fluid was administered during the surgery, all plasma mtDNA results were corrected based on the Hct measured on admission. As shown in Fig. 1, plasma mtDNA levels elevated significantly after the end of CPB (T2) and peaked at 12 h post-CPB (T4), which decreased remarkably at 24 h post-CPB (*P* < 0.05).

### Changes of perioperative cytokines levels

**Fig. 2:**
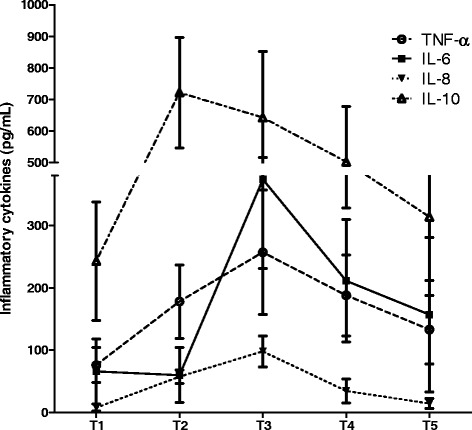
Merged curves of perioperative inflammatory cytokines in CABG with CPB. All inflammatory cytokines were elevated significantly after CPB (*P* < 0.05). Levels of TNF-α, IL-6 and IL-8 peaked at T3 while levels of IL-10 peaked at T2 (*P* < 0.05)

TNF-α, IL-6, IL-8 and IL-10 were measured by specific ELISA kits at the same time points. All concentrations were corrected based on the Hct measured on admission. Merged curves of these four cytokines were presented in Fig. 2. We found that all these cytokines but IL-6 began to increase significantly after T2 (*P* < 0.05). However, the highest concentrations of TNF-α and IL-8 appeared at T3, while IL-10 climbed the peak value at T2 (*P* < 0.05). Concentrations of IL-6 didn’t demonstrate a significant different at T2 comparing with T1 (*P* > 0.05). Interestingly, it peaked dramatically at T3 and then decreased gradually (*P* < 0.05).

### Correlations between plasma mtDNA and cytokines

**Fig. 3:**
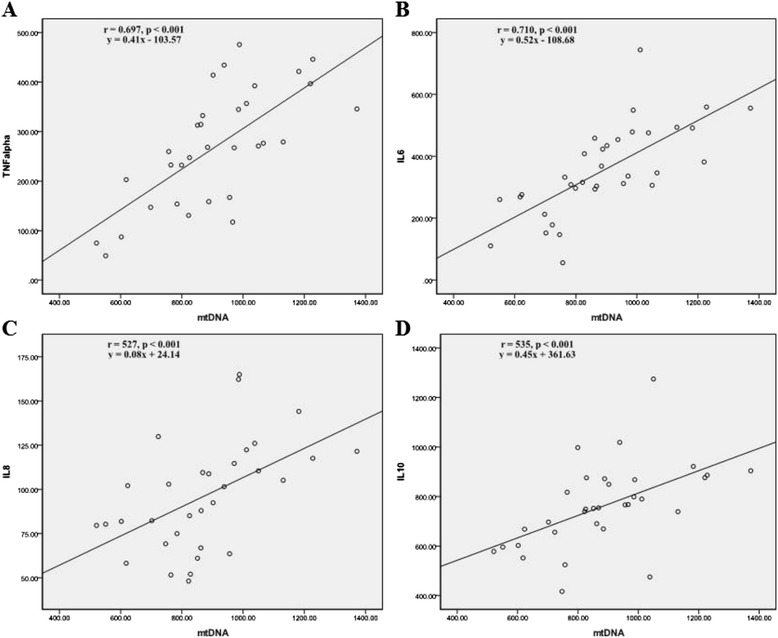
Correlation between the peak plasma mtDNA and peak inflammatory cytokines. **a** Scatter graph and the bivariate analysis demonstrated positive correlation between the peak plasma mtDNA and the peak TNF-α (*r* = 0.697, *P* < 0.001, *y* = 0.41*x* – 103.57). **b** Scatter graph and the bivariate analysis demonstrated positive correlation between the peak plasma mtDNA and the peak IL-6 (*r* = 0.710, *P* < 0.001, *y* = 0.52*x* – 108.68). **c** Scatter graph and the bivariate analysis demonstrated positive correlation between the peak plasma mtDNA and the peak IL-8 (*r* = 0.527, *P* < 0.001, *y* = 0.08*x* + 24.14). **d** Scatter graph and the bivariate analysis demonstrated positive correlation between the peak plasma mtDNA and the peak IL-10 (*r* = 0.535, *P* < 0.001, *y* = 0.45*x* + 361.63)

Bivariate correlations analysis were used to study the correlation between peak plasma mtDNA levels and peak cytokines levels. Positively correlation between the peak plasma mtDNA and the peak TNF-α (*r* = 0.697, *P* < 0.001), the peak IL-6 (*r* = 0.710, *P* < 0.001), the peak IL-8 (*r* = 0.527, *P* < 0.001) and the peak IL-10 (*r* = 0.535, *P* < 0.001) were confirmed and presented in Fig. 3.

## Discussion

Our study demonstrated that plasma mtDNA was released during cardiac surgery with CPB. We found the peak value of plasma mtDNA was shown up at 12 h post-CPB through five time points tracking analysis. Meanwhile, we confirmed releasing patterns of inflammatory cytokines, such as TNF-α, IL-6, IL-8 and IL-10, after cardiac surgery with CPB. We revealed a significantly and positively correlation between the peak plasma mtDNA and peak cytokines levels.

CPB-induced systemic inflammatory response syndrome was affected by many factors, such as the contact between blood and foreign surface of the extracorporeal circuits, hypothermia, reduction of pulmonary blood flow, direct surgical damage to the heart and endotoxemia [15]. It is studied that excessive inflammatory responses might delay the recovery and attenuate hospital outcomes [16]. As many studies reported, post-operative inflammatory responses were almost happened in every cardiac surgery with CPB, characterized with elevated inflammatory cytokines [6, 17]. In addition, levels of TNF-α, IL-6, IL-8 and IL-10, well-known inflammatory cytokines, represented the severity of inflammatory responses. Dr. Mei YQ *et al* found that concentrations of plasma IL-6 and IL-8 might be able to evaluate the severity of SIRS after CABG and establish the prognosis [18]. In the present study, we measured inflammatory cytokine levels after CABG with CPB. We found that all these inflammatory cytokines increased significantly after surgery with CPB, which apparently demonstrated that post-operative inflammatory responses occurred. Except for IL-10, which peaking at the end of CPB, all other cytokines peaked at 6 h post-CPB and levels of IL-8 dropped dramatically after peak time while others decreased gradually. Given that CPB-induced I/R condition may cause inflammatory responses [19], we suggest that IL-10 may act as the factor indicating direct damage-caused inflammatory responses while TNF-α, IL-6, IL-8 may represent I/R injury-induced inflammatory responses.

It is well studied that mtDNA was released after trauma surgery and the release of mtDNA happened in a cell necrosis-independent way. Additionally, the study also revealed that levels of plasma mtDNA were positively correlation with the invasiveness and the complexation of surgery [20]. As a pro-inflammatory agent, mtDNA was studied in many different fields and was confirmed that it can cause inflammatory responses [21–23]. Dr. Zhang Q *et al.* revealed that circulating mtDNA, a kind of mitochondrial damage-associated molecular patterns, can cause inflammatory responses and create a sepsis-like state [13]. In our study, after CABG with CPB, we found that plasma mtDNA levels elevated after the end of CPB and climbed to the peak value at 12 h post-CPB. And then it decreased gradually but still higher than the baseline level. It is reported that mtDNA was continuously released for at least five days after surgery [20]. As mentioned above, CPB, act as an I/R condition, can cause inflammatory responses. Combing with our result, plasma mtDNA elevation might indicate I/R injury-induced inflammatory responses. After the end of CPB, post-ischemia reperfusion caused secondary damage to the heart and late-peaked plasma mtDNA may indicate this secondary I/R injury-induced damage.

It is studied that plasma mtDNA can be a promising predictor for ICU stay and 28-day mortality [14]. In our study, the bivariate correlations analysis demonstrated a surprising positively correlation between the peak plasma mtDNA and the peak inflammatory cytokines. It is well known that excessive production of inflammatory cytokines may be a predictor for outcomes in different diseases and treatments [24, 25]. Considering that positively correlation between plasma mtDNA and inflammatory cytokines, we suggested that mtDNA might be involved in the progression of post-operative inflammatory responses.

## Conclusions

Our study found that release of mtDNA after cardiac surgery with CPB and positively correlation between plasma mtDNA and inflammatory cytokines, suggesting that mtDNA may play an important role in inflammatory responses after CPB. Our data strongly recommended a new insightful point at attenuating post-operative inflammation, which need further studies to illustrate more details about mtDNA-relative mechanism and effects. Additionally, considering that peak time of plasma mtDNA was 12 h post-CPB, later than other inflammatory cytokines, we need further studies to figure out the interaction between mtDNA and other inflammatory cytokines.
